# Sex and Strain Differences in Analgesic and Hyperlocomotor Effects of Morphine and μ‐Opioid Receptor Expression in Mice

**DOI:** 10.1002/jnr.70039

**Published:** 2025-04-18

**Authors:** Damien C. Boorman, Simran K. Rehal, Maryam Fazili, Loren J. Martin

**Affiliations:** ^1^ Department of Psychology The University of Toronto Mississauga Mississauga Ontario Canada; ^2^ Department of Cell and Systems Biology University of Toronto Toronto Ontario Canada

**Keywords:** DeepLabCut, locomotor activity, opioid, western blot, μ‐opioid receptor

## Abstract

Sex and gender differences in the analgesic efficacy and side effects of opioids have been widely reported, but their underlying neurobiological mechanisms remain poorly understood. Preclinical animal models are essential tools for investigating these differences and providing insights into the neurobiology of opioid effects. Although studies in rats have revealed sex‐specific effects of opioids, the sex‐dependent behavioral profiles of opioids in mice, particularly across strains, remain largely unexplored. In this study, we characterized sex and strain differences in the antinociceptive and hyperlocomotor effects of morphine in the two most widely used mouse strains—CD1 and C57BL/6—and quantified regional expression of the μ‐opioid receptor (MOR) in key brain and spinal cord regions. Both strains exhibited clear, dose‐dependent antinociceptive and hyperlocomotor responses to morphine. While no significant sex or strain differences were observed in antinociceptive effects, C57BL/6 mice displayed significantly greater hyperlocomotor activity than CD1 mice. Western blot analyses revealed strain‐specific MOR expression, with CD1 mice showing higher spinal cord and periaqueductal gray MOR levels, particularly in females, while C57BL/6 mice exhibited elevated MOR expression in the caudoputamen. Morphine treatment increased spinal MOR expression in CD1 mice but not C57BL/6, suggesting strain‐dependent regulation of MOR. These findings highlight strain‐specific behavioral and molecular responses to morphine, emphasizing the importance of strain and sex considerations in preclinical opioid research.

## Graphical abstract

Morphine dose‐dependently increased analgesia in both CD‐1 and C57BL/6 mice, with no observed sex differences. However, C57BL/6 mice of both sexes exhibited greater morphine‐induced locomotor activity compared to CD‐1 mice. These behavioral differences were paralleled by strain‐specific patterns of μ‐opioid receptor (MOR) expression: CD‐1 mice showed elevated MOR levels in the spinal cord and periaqueductal gray, whereas C57BL/6 mice had increased MOR expression in the caudoputamen.

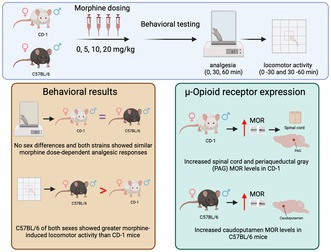

**Summary**

- This study highlights sex‐ and strain‐specific differences in morphine responses, underscoring the importance of sex and genetic background in understanding opioid effects.
- Our findings reveal different morphine‐induced behavior profiles and mu‐opioid receptor expression across two widely used mouse strains, CD1 and C57BL/6, and provide further insights into the neurobiological basis of opioid pharmacodynamics.
- These results emphasize the need to account for genetic and sex‐based variability for preclinical studies that seek to develop more effective and personalized opioid therapies for pain conditions.

## 1 Introduction

Chronic pain remains a pervasive and debilitating condition, affecting an estimated 20% of people worldwide, with the prevalence and burden of chronic pain disproportionately higher in women. Both human and animal research reports that women and females possess greater risks of developing chronic pain conditions (Riley et al. 1998). Additionally, a majority of studies report lower pain thresholds and tolerance in women (Sherman and LeResche 2006; Unruh 1996), indicating sex and gender differences in nociceptive responses and endogenous pain modulation (Berkley 1997; Fillingim and Ness 2000; Greenspan et al. 2007).

Opioids remain one of the most effective treatments for chronic pain and continue to be widely used, even in the face of well‐documented risks of addiction, tolerance, and overdose (Brookoff 2000; Dowell and Haegerich 2016). Contributing to the sex and gender disparity in the burden of pain conditions is evidence that opioids produce less analgesia and lead to more severe side effects—such as nausea, respiratory depression, and opioid‐induced hyperalgesia—in women compared to men (Fillingim 2005; Fillingim et al. 2009; Miller and Ernst 2004; Mogil and Bailey 2010). However, the neurobiological mechanisms underlying these sex and gender differences in opioid efficacy and side effects remain poorly understood.

Preclinical animal models have been, and continue to be, indispensable tools for uncovering the neurobiological underpinnings of these phenomena, enabling the investigation of sex‐specific effects in a controlled setting. Prior rodent studies, mainly using rats, have found significant sex differences in the antinociceptive and side‐effect profiles of opioids. For example, morphine—the most widely studied opioid—consistently demonstrates greater potency and efficacy in male rodents, while females often require higher doses to achieve comparable analgesia. Moreover, female rodents exhibit more rapid tolerance and greater susceptibility to opioid‐induced side effects such as hyperalgesia and respiratory depression (Peckham and Traynor 2006; Stoffel et al. 2005; Terner et al. 2003).

Understanding the neurobiological mechanisms that drive sex differences in the processing of opioids would allow for the development of more targeted and potentially sex‐specific therapies. Opioids mediate their antinociceptive effects primarily through binding to the μ‐opioid receptor (MOR), a G‐protein‐coupled receptor highly expressed throughout pain‐processing regions in the brain and spinal cord (Bernal et al. 2007; Loyd et al. 2008; Peckham and Traynor 2006). Specifically, MOR activation inhibits neuronal excitability by reducing the release of neurotransmitters, thereby dampening nociceptive signaling.

Emerging evidence suggests that differences in MOR expression, distribution, and function across specific brain regions may drive variability in opioid efficacy and side effects. Indeed, studies in both humans and rodents have identified sex differences in MOR expression in some of these key brain regions, including the midbrain, amygdala, and anterior cingulate cortex (Loyd et al. 2008; Wang et al. 2020; Won et al. 2023). However, much of this work has focused on rats, even though mice have become the predominant model in preclinical research due to their genetic manipulability and the availability of transgenic tools. Extending these investigations to mice would enable more powerful functional studies to uncover the mechanisms driving these sex differences.

Despite the utility of mouse models, sex and strain differences in the behavioral and molecular effects of opioids in mice remain poorly understood. Furthermore, most preclinical studies investigating pain and analgesia have historically been conducted exclusively in males, with approximately 79% of preclinical pain studies excluding females (Mogil 2012). To address these gaps, the current study aimed to characterize and compare sex differences in the antinociceptive and side effect profiles of morphine and to quantify regional MOR expression in the spinal cord and pain‐related brain regions. These analyses were conducted in the two most commonly used mouse strains in preclinical research, CD1 and C57BL/6, providing a comprehensive assessment of sex‐ and strain‐specific responses to morphine.

## 2 Materials and Methods

### 2.1 Experimental Design

**FIGURE 1:**
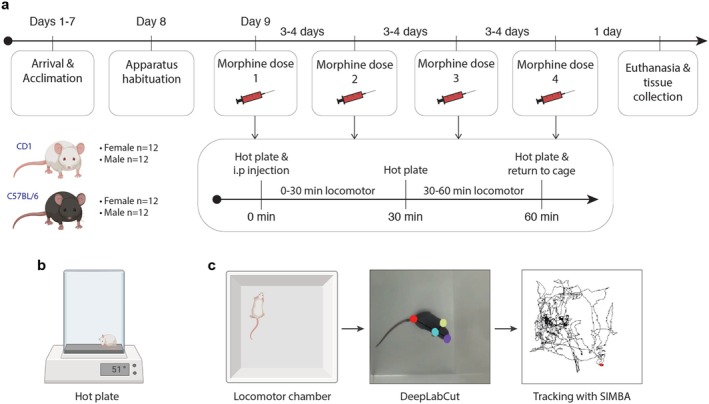
Experimental design. 6‐ to 10‐week‐old CD1 (*n* = 12 F, 12 M) and C57BL/6 mice (*n* = 12 F, 12 M) were used for these experiments. (a) Experimental timeline. After acclimation to laboratory conditions and apparatus habituation, mice received four doses of morphine (0, 5, 10, and 20 mg/kg i.p) in a counterbalanced order with 3‐ to 4‐day washout periods between each dose. For each dose, pain thresholds and antinociception were measured using (b) a hot plate (45 s at 51°C) 0‐, 30‐, and 60‐min post‐injection. (c) Locomotor activity was measured in two periods (0–30 and 30–60 min post‐injection) by placing mice into an open arena (15 × 15 cm) between hot plate testing. The open‐sourced software DeepLabCut and Simple Behavioral Analysis (SIMBA) were used to quantify the distance traveled by each mouse.

This study aimed to investigate sex and strain differences in the antinociceptive and side‐effect profiles of morphine in two of the most commonly used strains of mice in preclinical research—CD1 and C57BL/6. We used a standard hot plate test to quantify pain thresholds and antinociception by measuring the latency to the first pain response. To assess morphine‐induced side effects, we measured hyperlocomotor activity—the most well‐known side effect produced by opioids in rodents (Figure 1). Dose–response curves for both antinociception and hyperlocomotor activity were calculated using 4 doses of morphine—0, 5, 10, and 20 mg/kg. An experimenter, blinded to drug treatment, performed behavioral testing on each day. We then investigated the possible neurobiological bases of sex and strain differences by quantifying μ‐opioid receptor (MOR) protein expression in 5 brain regions involved in processing pain and/or locomotion. These were the spinal cord (pain and locomotion), the periaqueductal gray (pain), the caudoputamen (locomotion), the nucleus accumbens (pain and locomotion), and the anterior cingulate cortex (pain).

### 2.2 Animals and Housing

Forty‐eight 6‐ to 10‐week‐old C57BL/6J (*n* = 12 M, 12 F) and CD‐1 (*n* = 12 M, 12 F) mice obtained from Charles Rivers Laboratories (Wilmington, MA, USA) were used for these experiments. Mice were housed individually in ventilated cages in a temperature‐controlled environment (20°C ± 1°C) under a 12‐h light/dark cycle (lights ON at 7 am) with *ad libitum* access to standard chow and water. A compressed cotton nesting square and crinkled paper bedding were provided in each cage as a source of environmental enrichment. Procedures followed the animal care standards set forth by the Canadian Council on Animal Care (CCAC) and approved by the University of Toronto's Biological Sciences Animal Care Committee. The principles of the three R's (replacement, reduction, and refinement) were strictly applied to minimize pain and discomfort, and the study adhered to the IASP's ‘Ethical Guidelines for Investigations of Experimental Pain in Conscious Animals’ (Zimmermann 1983).

### 2.3 Drug Administration

Morphine (CDMV, Saint‐hyacinthe, Quebec, Canada) was freshly prepared and diluted with 0.9% saline within 1 h of administration. Four doses of morphine (0, 5, 10, and 20 mg/kg) were administered to each mouse (10 mL/kg, i.p.) in a randomized and counterbalanced order over a 2‐week period. Morphine was administered with a washout period of 3–4 days between each subsequent dose to ensure no residual effects from prior administrations. Saline controls never received morphine.

### 2.4 Hot Plate Test

Mice were placed on a hot plate set at 51.0°C (±0.1°C variance) in a 15 [w] × 15 [d] × 40 cm [h] acrylic chamber for 45 s, and the latency to perform a hind paw withdrawal, hind paw lick, or hind paw shake was recorded. Mice were tested on the hot plate immediately before drug administration (0 min), then 30‐ and 60‐min post‐injection. Each test lasted 45 s to avoid tissue damage, with all animals remaining on the hot plate for the 45 s to avoid animals learning that paw withdrawal and licking responses are associated with their removal from the hot plate.

### 2.5 Locomotor Measurement

Locomotor behavior was measured for two intervals following morphine or saline administration: 0–30 and 30–60 min post‐injection. Immediately after the 0‐ and 30‐min hot plate tests, each mouse was placed into a 15 [w] × 15 [d] × 40 cm [h] acrylic chamber. Mice were allowed to move freely, and their movements were digitally recorded from above. The total distance traveled by each mouse for each interval was calculated using the open‐sourced software DeepLabCut and Simple Behavioral Analysis (SIMBA), which tracked the movement of the base of the mouse's tail.

### 2.6 Western Blotting

Twenty‐four hours after the last test, mice were cervically dislocated and rapidly decapitated. Brains and spinal cords were immediately extracted and snap‐frozen with liquid nitrogen and stored at −80°C until required. Microdissections of brain regions took place on ice, and the isolated brain regions were weighed to ensure consistency and then stored at −20°C. Tissue was homogenized in radioimmunoprecipitation assay (RIPA) buffer (Millipore Sigma 20‐188) containing protease and phosphatase inhibitors (ThermoFisher Scientific, 78440), then centrifuged at 14,000 rpm for 20 min at 4°C. The supernatant containing total protein was then collected into new Eppendorf tubes. Protein concentrations were determined using the Bradford assay, following the manufacturer's instructions (BioRad Quick Start, Bradford 1× Dye Reagent, 5000205).

Next, equal amounts of protein (20 μg) were loaded onto 10% SDS‐polyacrylamide gels (TGX FastCast Acrylamide Starter Kit, 10%, 1610172) and electrophoresed (BioRad PowerPac HC High‐Current Power Supply, 1645052; Mini‐PROTEAN Tetra Vertical Electrophoresis Cell for Mini Precast Gels, 4‐gel, 1658004) at a constant voltage of 250 V for 30 min or until the dye front reached near the bottom of the gel. Proteins were transferred to a polyvinylidene fluoride (PVDF) membrane using a wet transfer system (Mini Trans‐Blot Module, 1703935).

For immunohistochemistry, the membrane was first blocked in 5% skimmed milk in Tris‐buffered saline with 0.1% Tween‐20 (TBS‐T) and rocked for 1 h at room temperature. Membranes were then co‐incubated overnight at 4°C with primary antibodies targeting the μ‐opioid receptor (MOR) (Millipore Sigma, AB1580‐I; RRID:AB_2716850) and β‐actin (Sigma‐Aldrich, A5316; RRID:AB_476743) at a 1:5000 dilution in blocking buffer (3% BSA in Tris‐buffered saline with 0.1% Tween‐20 (TBS‐T)). This MOR antibody produces several bands, including at known sizes of MOR isoforms, with the strongest at 45–50 and 65–70 kDa, which we quantified. This antibody has been used in several previous studies (Hoover et al. 2021; Vicario et al. 2022, 2019; Zhu et al. 2018), supporting its specificity. However, additional validation, such as testing in MOR knockout mice, would further confirm its selectivity and rule out potential off‐target binding. Following primary antibody incubation, membranes were washed with TBS‐T (3 × 5 min) and then incubated with HRP‐conjugated secondary antibody (anti‐rabbit; Cedarlane Labs, 711‐035‐152) and rocked at room temperature for 1 h before imaging. Subsequently, membranes were incubated with a second HRP‐conjugated secondary antibody (anti‐mouse; Cedarlane Labs, 515‐035‐062), again rocked for 1 h at room temperature, and then imaged.

For imaging, a chemiluminescent substrate (Thermo Scientific, SuperSignal West Femto Maximum Sensitivity Substrate, 0034095) was applied to the membrane for 2 min to visualize the protein bands, and signals were captured using a chemiluminescence imaging system (iBiright 1500; iBright 750). Two main bands were quantified at ~50 and ~70 kDa. It has been previously found that the MOR undergoes alternative splicing, producing multiple isoforms with distinct structural and functional properties (Liu et al. 2021). The 50 kDa MOR isoform represents the canonical 7‐transmembrane receptor, primarily coupling to Gαi/o proteins to mediate classic opioid‐induced analgesia and inhibition of adenylyl cyclase, with high affinity for morphine (EC50 ~ 17.9 nM) (Huang et al. 2015; Zhang et al. 2022). In contrast, the 70 kDa MOR isoform is likely a glycosylated form or splice variant with altered signaling properties, potentially engaging different G proteins and exhibiting distinct phosphorylation patterns, though its precise role in morphine binding and downstream effects remains less well characterized (Liu et al. 2021). Band intensities of each isoform were quantified with ImageJ using the mean gray method. First, the intensity of each band was measured, and the background immediately above and below was subtracted to calculate the signal. The MOR signal was normalized to the signal of the beta‐actin band and then expressed as a ratio to a pooled sample, which contained samples from all mice in the study.

### 2.7 Statistical Analysis

Statistical tests were performed in either GraphPad Prism v9.0.1 (San Diego, California, USA) for two‐way ANOVAs and linear regressions or in R v3.6.1 (R Core Team 2021) using RStudio v1.4.1106 for three‐way ANOVAs. For all statistical tests, *α* = 0.05. To assess sex and strain differences in baseline hot plate and locomotor behaviors, three‐way ANOVAs (sex*strain*time) were computed for each data set. Likewise, three‐way, repeated measures ANOVAs were computed to assess sex and strain differences in dose–response curves for morphine‐induced antinociception and hyperlocomotor effects (sex*strain*dose). Post hoc multiple pairwise comparisons to assess sex and strain differences at each dose were performed in R using the emmeans package (v1.5.5‐1; Lenth 2021), with Tukey's correction. Simple linear regressions were run to assess correlations between antinociceptive and hyperlocomotor effects. Two‐way ANOVAs were computed for each isoform in each region to assess sex and strain differences in MOR expression. Two‐way ANOVAs were also computed (treatment*strain) for each isoform in each region, comparing MOR expression between saline and morphine‐treated animals. For full details and results of statistical testing, see Table S1.

## 3 Results

### 3.1 No Sex or Strain Differences in the Antinociceptive Effects of Morphine

**FIGURE 2:**
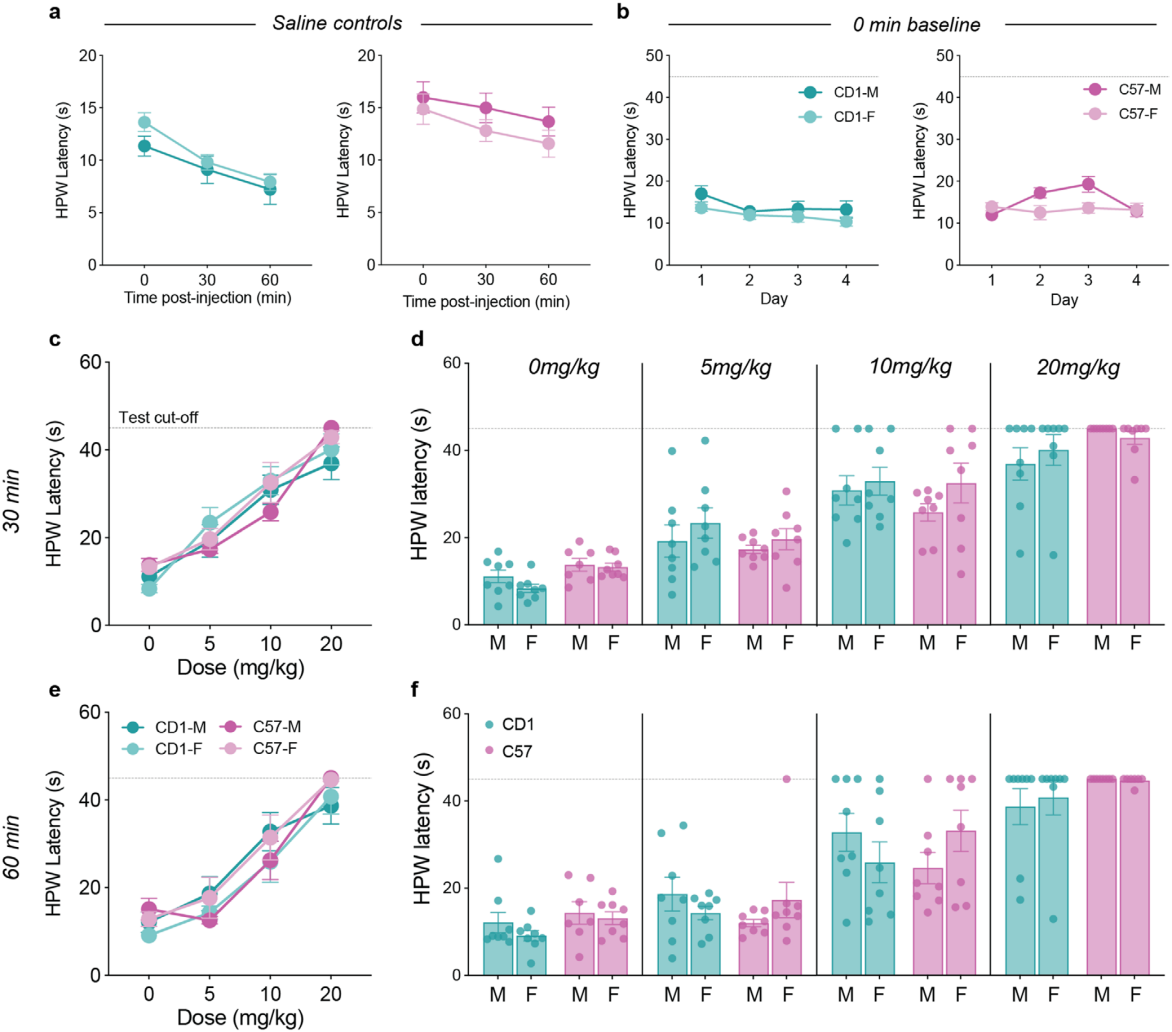
Pain thresholds and morphine‐induced antinociception. (a) Hind paw withdrawal latency (HPWL) in saline controls reduced over the 60‐min testing period in both strains. CD1s tended to have shorter HPWL than C57s but with no sex differences. (b) All mice showed similar HPWLs at the 0‐min baseline test across all four testing days, though again, with small reductions across time. (c, d) Morphine dose‐dependently increased HPWLs at the 30‐min post‐injection test for all mice. (e, f) The same pattern was observed for the 60‐min post‐injection hot plate test. Three‐way, repeated measures ANOVAs revealed that there were no significant sex or strain differences observed at either timepoint in morphine‐induced antinociception at any of the doses given.

Across the 60‐min testing period, hind paw withdrawal latency (HPWL) in saline‐treated control mice decreased progressively in both CD1s and C57s, indicating a time‐dependent reduction in withdrawal latency under repeated hot plate exposure. CD1 mice exhibited consistently shorter HPWLs compared to C57 mice, suggesting strain differences in baseline thermal sensitivity. No significant sex differences were observed for either strain (Figure 2a). As such, a three‐way, repeated measures ANOVA found a significant main effect of time (*F*_2,24_ = 26.66, *p* < 0.0001) and strain (*F*_1,12_ = 8.42, *p* = 0.013) but no interactions between any of the factors. Interestingly, a three‐way, repeated measures ANOVA of the baseline HPWLs (0‐min) across the four testing days found no significant main effects or interaction effects between sex, strain, and time. This suggests that the effect of repeated testing on reducing HPWLs was an acute effect that did not persist across time after the 3‐ to 4‐day washout period (Figure 2b).

Morphine consistently and dose‐dependently increased hind paw withdrawal latency at both 30‐ and 60‐min post‐injection. Notably, the majority of mice treated with the 20 mg/kg dose reached the test cut‐off time of 45 s (Figure 2c–f). However, there were no sex or strain differences in these antinociceptive effects. As such, for both the 30‐ and 60‐min tests, three‐way, repeated measures (sex*strain*dose) ANOVAs found significant main effects of dose (*F*_3,81_ = 108.0, *p* < 0.0001; *F*_3,81_ = 82.1, *p* < 0.0001), but no main effects of sex (*F*_1,27_ = 0.89, *p* = 0.35; *F*_1,27_ = 0.003, *p* = 0.96), or strain (*F*_1,27_ = 0.28, *p* = 0.60; *F*_1,27_ = 0.70, *p* = 0.41), nor any interaction effects between these factors (see Table S1 for full results of statistical analyses).

### 3.2 Strain, but No Sex Differences, in the Hyperlocomotor Effects of Morphine

**FIGURE 3:**
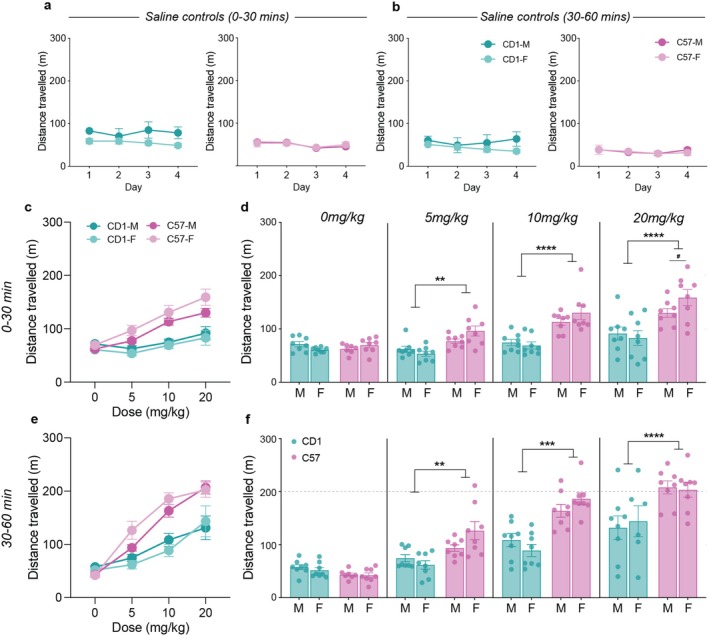
Locomotor and morphine‐induced hyperlocomotor activity. Baseline locomotor activity for the saline controls during the (a) 0–30 min and (b) 30–60 min testing periods. No significant sex or strain differences were observed. (c, d) Dose–response curves for morphine‐induced hyperlocomotor activity for both sexes and strains at 0–30 min. Morphine produced significantly stronger hyperlocomotor effects in C57s at all doses given. No sex differences were observed. (e, f) The same pattern of effects was observed for the 30–60 min period. **p < 0.01, ***p < 0.001, ****p < 0.0001, three‐way, repeated measures ANOVAs.

Mice that only received saline showed consistent levels of locomotor activity across the 4 testing days (Figure 3a,b). A strain difference, but no sex difference, was observed, with CD1s showing slightly greater locomotor activity than C57. This effect was more pronounced in the 0‐ to 30‐min interval. As such, for this interval, a three‐way, repeated measures (sex*strain*time) ANOVA found a significant effect of strain (*F*_1,11_ = 8.33, *p* = 0.015) but not sex (*F*_1,11_ = 3.63, *p* = 0.083) or time (*F*_3,33_ = 1.24, *p* = 0.30) and no interaction effects between these factors. No significant main or interaction effects were found at the 30‐ to 60‐min interval.

Similar to the morphine‐induced antinociceptive effects, at both the 0‐ to 30‐ and 30‐ to 60‐min intervals, morphine clearly, reliably, and dose‐dependently increased locomotor activity. However, unlike the antinociceptive effects, we observed a large strain difference, with C57s showing significantly greater locomotor activity than CD1s at all doses (Figure 3c–f). Supporting these observations, three‐way, repeated measures ANOVAs found main effects of strain and dose for both the 0‐ to 30‐min interval (*F*_1,27_ = 43.12, *p* < 0.0001; *F*_3,81_ = 31.27, *p* < 0.0001) and the 30‐ to 60‐min interval (*F*_1,27_ = 22.32, *p* < 0.0001; *F*_3,81_ = 87.95, *p* < 0.0001). Additionally, a significant sex*strain interaction effect was found for the 0‐ to 30‐min interval (*F*_1,27_ = 6.64, *p* = 0.016), but post hoc multiple comparisons only found a statistically significant sex difference for C57s at the 20 mg/kg dose, with females showing greater locomotor activity than males (*p* = 0.032).

### 3.3 Correlations Between Morphine‐Induced Antinociception and Side‐Effects

**FIGURE 4:**
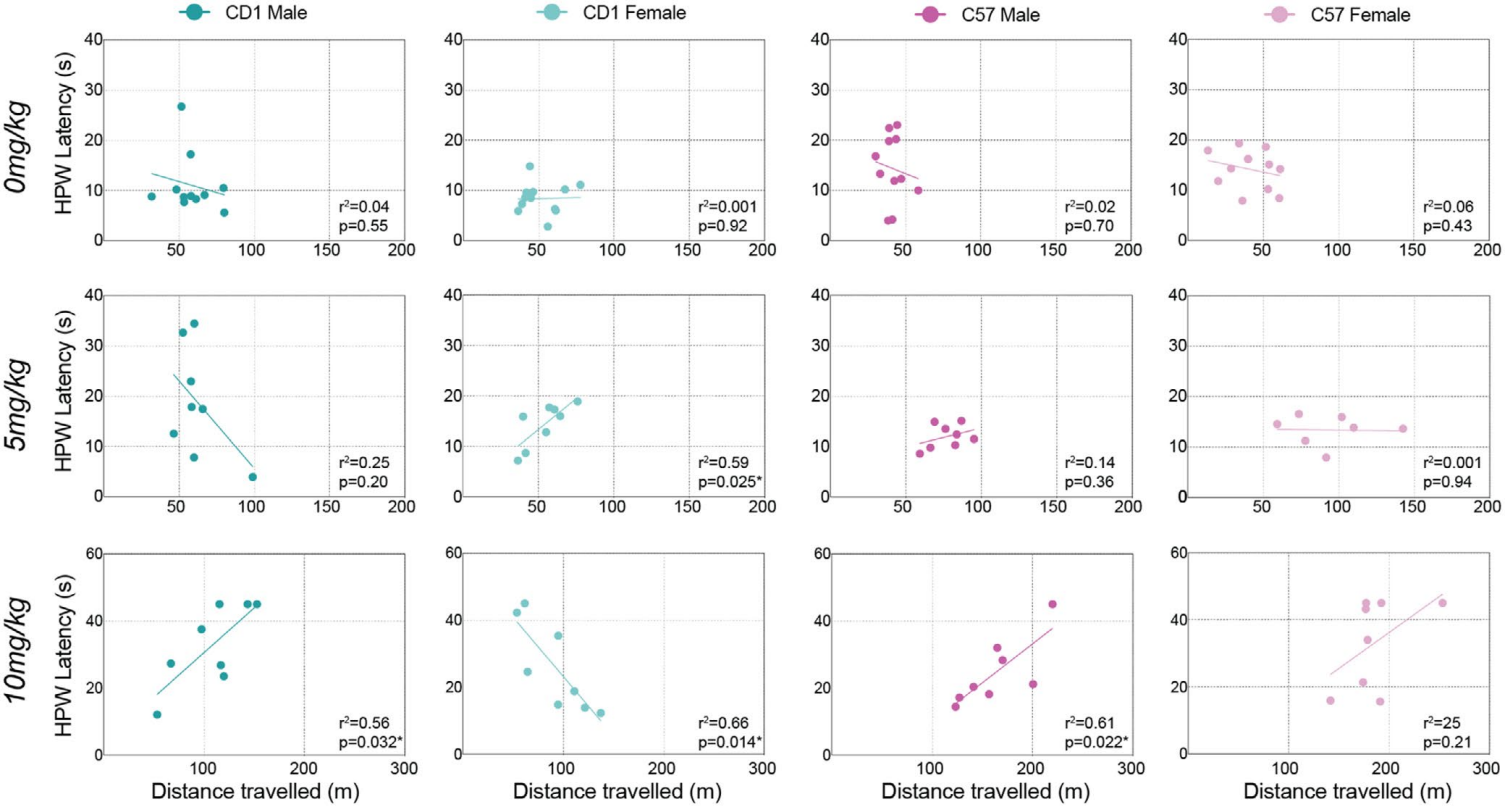
Correlation between morphine‐induced antinociception and hyperlocomotor activity. Linear regressions between HPWL at 60 min post‐injection and distance traveled during the 30–60 min period revealed sex‐ and strain‐specific effects. At the 0 mg/kg dose (i.e., saline), hind paw withdrawal latency and locomotor activity were not correlated, indicating that these two behaviors are generally independent of each other (also see Figure S1). At 5 mg/kg, only CD‐1 females showed a positive correlation between these measures. In contrast, at 10 mg/kg, a negative correlation was observed for CD1 females, while positive correlations were seen for CD1 males and C57 males and females. Linear regressions were not run for 20 mg/kg due to the ceiling effects of morphine on HPWL.

Linear regressions were run between the distance traveled during the 30‐ to 60‐min post‐injection interval and the HPWL on the 60‐min post‐injection hot plate test to assess whether antinociceptive and hyperlocomotor effects were correlated. These timepoints were chosen to coincide with the peak antinociceptive and hyperlocomotor effects of morphine. Correlations at 20 mg/kg were not run due to ceiling effects in antinociception at this dose (i.e., most mice reached the cut‐off time of 45 s). We found an interesting pattern of correlations (Figure 4). Firstly, at the 0 mg/kg dose (i.e., saline), hind paw withdrawal latency and locomotor activity were not correlated, indicating that these two behaviors are generally independent of each other (also see Figure S1). At the 5 mg/kg dose, female CD1s showed a significant positive correlation between these effects (*r* ^2^ = 0.59, *p* = 0.025), with the CD1 males and both sexes of C57s showing no significant correlations. In contrast, at the 10 mg/kg dose, female CD1s showed a significant negative correlation between these effects (*r* ^2^ = 0.66, *p* = 0.014), while male CD1s (*r* ^2^ = 0.56, *p* = 0.032) and male C57s (*r* ^2^ = 0.61, *p* = 0.022) showed positive correlations. Female C57s showed a similar trend line as the males of both sexes, but this correlation was not statistically significant (*r* ^2^ = 0.25, *p* = 0.21).

### 3.4 μ‐Opioid Receptor (MOR) Expression in the Spinal Cord, Periaqueductal Gray (PAG), Caudoputamen, Nucleus Accumbens (NAc), and the Anterior Cingulate Cortex (ACC)

Next, we quantified MOR protein expression using western blotting in the spinal cord and several brain regions known to be involved in pain processing, antinociception, and/or locomotion to investigate any sex and strain differences that could contribute to the behavioral differences in morphine‐induced effects. We quantified the two most abundant MOR isoforms (~70 and ~50 kDa). Additionally, to determine whether morphine administration could alter MOR expression in these regions, we compared the mice that received the three doses of morphine to the saline controls.

**FIGURE 5:**
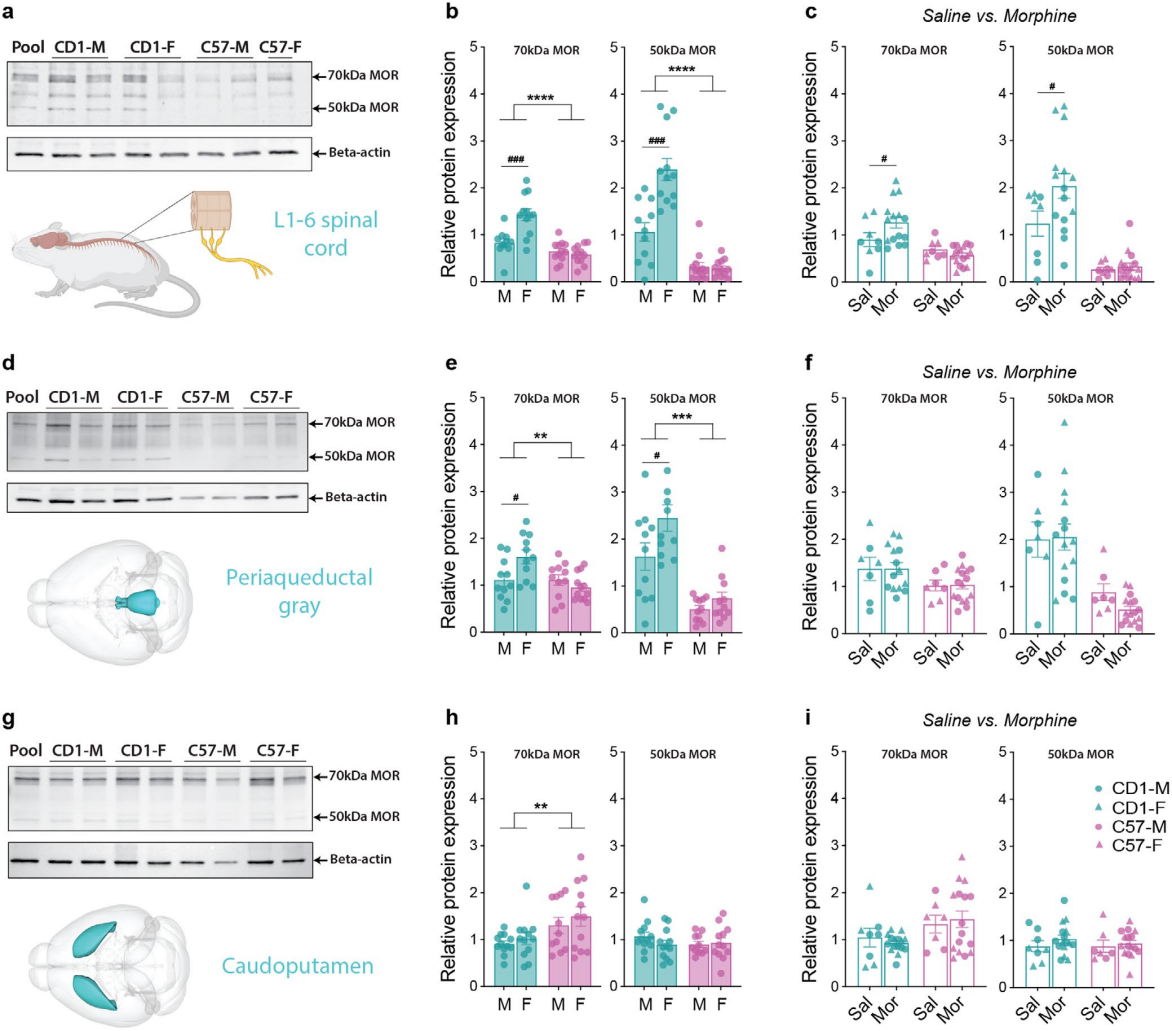
μ‐Opioid receptor (MOR) expression in the spinal cord, periaqueductal gray, and caudoputamen. (a) Representative western blot of MOR and beta‐actin expression in the L1‐6 spinal cord, showing the 70 and 50 kDa isoforms of MOR. (b) CD1 mice had greater MOR expression than C57 mice for both isoforms. For CD1s, females had greater MOR expression than males, but no sex differences were observed for C57s. (c) Administration of the three doses of morphine increased MOR expression in the spinal cord for CD1s but not C57s. No sex differences were observed, so data from males and females were pooled for this analysis. (d) Representative western blot of MOR and beta‐Actin expression in the periaqueductal gray. (e) Similar to the spinal cord, CD1s showed greater MOR expression than C57s, and for CD1s, females showed higher expression than males. (f) Administration of morphine had no effect on MOR expression in PAG in either strain. (g) Representative western blot of MOR and beta‐actin expression in the caudoputamen. In contrast to the spinal cord and the PAG, C57s had greater MOR expression in the 70 kDa isoform (but not the 50 kDa isoform). No sex differences were observed. (i) Morphine administration did not affect MOR expression in the caudoputamen in either strain. Two‐way (sex*strain) ANOVAs with post hoc multiple comparisons tests (Tukey's correction). For Stain comparisons, **p < 0.01, ***p < 0.001, ****p < 0.00001. For within group (sex or drug) comparisons, #*p* < 0.05, ###*p* < 0.001.

In the spinal cord, we found that CD1s had greater MOR expression than C57s and a strain‐specific sex difference, with females having greater MOR expression than males for only CD1s (Figure 5b). Supporting these observations, two‐way ANOVAs (sex*strain) found a significant interaction effect for both the 70 kDa (*F*_1,43_ = 15.28, *p* = 0.0003) and 50 kDa (*F*_1,43_ = 18.06, *p* = 0.0001) isoforms. Interestingly, CD1 mice, but not C57 mice, that received morphine had increased MOR expression in the spinal cord, with two‐way ANOVAs (strain*treatment) finding a significant interaction effect for the 70 kDa isoform (*F*_1,43_ = 5.29, *p* = 0.026) and a treatment effect for the 50 kDa isoform (*F*_1,43_ = 4.23, *p* = 0.046) (Figure 5c).

In the periaqueductal gray, we found a remarkably similar pattern of MOR expression as in the spinal cord, with CD1s showing greater MOR expression than C57s and only female CD1s showing greater expression than their male counterparts (Figure 5e). Two‐way ANOVAs (sex*strain) found a significant interaction effect for the 70 kDa isoform (*F*_1,41_ = 7.34, *p* = 0.009) and main effects for sex (*F*_1,41_ = 6.06, *p* = 0.018) and strain (*F*_1,41_ = 43.77, *p* < 0.0001) for the 50 kDa isoform, but no interaction effect (*F*_1,41_ = 1.92, *p* = 0.17). Morphine administration did not affect MOR expression in the PAG (Figure 5f).

In contrast to the spinal cord and the PAG, we found that C57s had greater MOR expression than CD1s in the caudoputamen, but only in the 70 kDa isoform. No sex differences in either strain were observed (Figure 5g). As such, a two‐way ANOVA (sex*strain) found a significant main effect of strain (*F*_1,43_ = 8.25, *p* = 0.006) but no main effect of sex (*F*_1,43_ = 1.02, *p* = 0.32) or an interaction effect (*F*_1,43_ = 0.062, *p* = 0.80). As with the PAG, morphine administration had no effect on MOR expression in the caudoputamen (Figure 5i).

**FIGURE 6:**
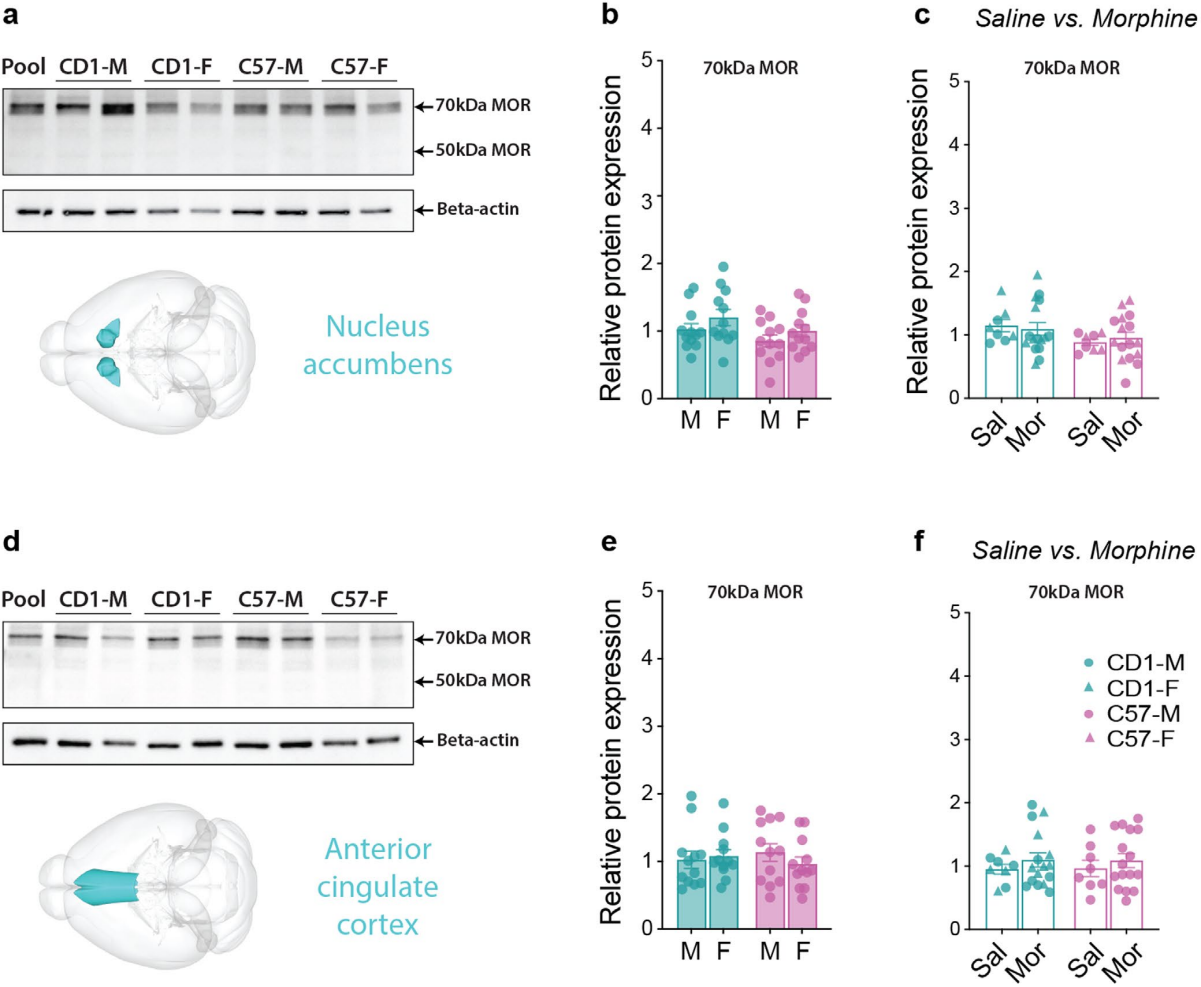
μ‐Opioid (MOR) receptor levels in the nucleus accumbens (NAc) and the anterior cingulate cortex (ACC). (a) The western blot of MOR and beta‐actin expression in the NAc shows the 70 and 50 kDa bands of MOR. The 50 kDa band was not quantified as there was negligible expression of this isoform in this region. (b) No sex or strain differences were observed in MOR expression in the NAc. (c) Morphine administration had no effect on MOR expression in the NAc. (d) Representative western blot of MOR and beta‐actin expression in the ACC. The 50 kDa band was not quantified, as this isoform was not expressed in this region. (e) No sex or strain differences were observed in MOR expression in the ACC. (f) Morphine administration had no effect on MOR expression in the ACC. Two‐way (sex*strain) ANOVAs.

Interestingly, in both the nucleus accumbens and the anterior cingulate cortex, only the 70 kDa isoform of MOR was expressed at quantifiable levels. In both of these regions, no sex or strain differences in MOR expression were observed (Figure 6b,e). Furthermore, morphine administration had no effect on MOR expression in either region (Figure 6c,f). Finally, we found that MOR expression was not impacted by which dose of morphine was given on the final day of testing (i.e., 24 h before euthanasia and tissue collection) (Figure S2).

## 4 Discussion

In this study, we systematically investigated sex‐ and strain differences in morphine's antinociceptive and side‐effect profiles, as well as μ‐opioid receptor (MOR) expression in the spinal cord and key pain‐ and locomotion‐related brain regions in the two most commonly used mouse strains, CD1 and C57BL/6. Our findings reveal nuanced behavioral and region‐specific effects, with significant implications for understanding the neurobiological underpinnings of opioid antinociception and side effects.

### 4.1 Strain‐Dependent Thermal Sensitivity and Morphine Antinociception

Baseline thermal sensitivity revealed apparent strain differences, with CD1 mice consistently exhibiting shorter hind paw withdrawal latencies (HPWL) compared to C57BL/6 mice. This finding aligns with previous studies showing strain variability in nociceptive thresholds and highlights the importance of genetic background in pain sensitivity (Lariviere et al. 2002; Mogil 2012; Smith 2019). Despite this baseline difference, morphine dose‐dependently increased HPWL across both strains, and no significant sex or strain differences were observed in the antinociceptive effects. This is consistent with earlier research demonstrating the robust efficacy of morphine in alleviating thermal nociception in both sexes (Celerier et al. 2006; Elhabazi et al. 2014). However, the absence of observed sex differences in morphine antinociception diverges from myriad reports in rats showing greater morphine potency in males (Bartok and Craft 1997; Boorman and Keay 2021; Cook et al. 2000; Gabel et al. 2023; Loyd et al. 2008). One possible explanation is species‐specific variability in MOR signaling or differences in study design, such as using different nociceptive assays or doses. Additionally, ceiling effects at higher doses (e.g., 20 mg/kg) may have masked subtle sex differences. Further studies employing alternative pain assays, pain measures, or sub‐antinociceptive doses may better elucidate sex‐specific antinociceptive effects in mice.

### 4.2 Strain‐Dependent Hyperlocomotor Effects of Morphine

A pronounced strain difference was observed in morphine‐induced hyperlocomotion, with C57BL/6 mice displaying significantly greater locomotor activity than CD1 mice across all doses and time intervals. This finding is consistent with prior reports of heightened sensitivity to opioid‐induced locomotor effects in C57BL/6 mice (Belknap et al. 1998; Bryant et al. 2009; Elmer et al. 2010). Interestingly, a sex–strain interaction effect was observed during the early (0–30 min) interval, where C57 females exhibited greater hyperlocomotor activity than males at the highest morphine dose (20 mg/kg). This finding adds to the growing evidence suggesting that genetic background and sex may interact to modulate opioid‐induced behaviors (Cook et al. 2000; Gabel et al. 2023; Loyd et al. 2008).

The dissociation between antinociceptive and hyperlocomotor effects observed in this study may reflect differential engagement of distinct MOR populations and downstream signaling pathways. Whereas morphine‐induced antinociception is mediated primarily through MOR activation in descending pain modulatory pathways, hyperlocomotion is thought to involve MORs in mesolimbic regions, such as the nucleus accumbens and ventral tegmental area (Amalric and Koob 1985; Funada et al. 1994; Severino et al. 2020; Tomsic and Maldonado 1990). Therefore, the observed strain differences may reflect divergent MOR expression or function in these circuits, as supported by our own MOR expression findings.

### 4.3 Correlations Between Antinociceptive and Locomotor Effects

Correlational analyses revealed intriguing strain‐ and sex‐specific patterns between morphine‐induced antinociception and hyperlocomotion. At lower doses (5 mg/kg), CD1 females exhibited a positive correlation between these effects, suggesting a linked neurobiological mechanism. However, this relationship inverted to a negative correlation at higher doses (10 mg/kg), while males of both strains maintained positive correlations. These findings suggest that the relationship between antinociceptive and hyperlocomotor effects may not only vary by sex and strain but also depend on dose and MOR signaling dynamics. One hypothesis is that competing MOR‐mediated processes, such as receptor desensitization or biased signaling, may differentially modulate these behaviors across experimental conditions (Bohn et al. 2000; Che et al. 2021).

### 4.4 MOR Expression and Potential Mechanisms

Consistent with previous studies, MOR expression varied significantly by sex, strain, and brain region (Guajardo et al. 2017; Loyd and Murphy 2014; Noble and Cox 1996; Xu et al. 2014). CD1 mice exhibited greater MOR expression than C57BL/6 mice in the spinal cord and periaqueductal gray (PAG); regions central to pain modulation (Heinricher et al. 2009; Hemington and Coulombe 2015; Ossipov et al. 2014). Moreover, a sex‐specific difference was observed in CD1 mice, with females showing higher spinal cord and PAG MOR expression compared to males, a finding in contrast with (Loyd et al. 2008), who found increased MOR expression in male versus female rats in the PAG using immunohistochemistry. Our findings suggest that higher MOR availability in CD1 females may enhance their sensitivity to endogenous or exogenous opioids in these regions, potentially explaining the observed correlation between antinociception and hyperlocomotion at lower doses.

However, the lack of corresponding sex and strain differences in antinociception despite large differences in MOR expression patterns implies the presence of compensatory mechanisms that mitigate this enhanced MOR expression at the behavioral level. These compensatory processes may include differences in receptor downstream signaling pathways, receptor desensitization, or interactions with other modulatory systems, such as endogenous opioid peptides or inhibitory neurotransmitters (Bohn et al. 2000; Vaughan et al. 1997). Such mechanisms could effectively normalize the antinociceptive response despite varying MOR activity. Alternatively, the differential activation of other brain regions or circuits outside the spinal cord and PAG may influence the overall pain experience, diluting the impact of MOR expression differences. This underscores the complexity of opioid antinociception and highlights the need for a more integrative approach to understanding how MOR expression translates or, in our case, fails to translate into behavioral outcomes.

In contrast to the spinal cord and PAG, C57BL/6 mice displayed greater MOR expression in the caudoputamen, a region implicated in locomotor activity (Fobbs et al. 2020; Kravitz et al. 2010; Yin 2017). The heightened hyperlocomotor effects observed in C57 mice may reflect greater MOR‐mediated activation of striatal circuits. Conversely, it is also possible that the increased MOR expression in the spinal cord of the CD1s could reduce the hyperlocomotor effects of morphine if their activation were to dampen down excitatory motor signals. Importantly, no sex differences in MOR expression were observed in the caudoputamen, consistent with the lack of robust sex differences in locomotor behavior across most doses.

Interestingly, no strain or sex differences in MOR expression were detected in the nucleus accumbens or anterior cingulate cortex (ACC), regions critical for opioid reward and cognitive aspects of pain, respectively (Becerra and Borsook 2008; Bushnell et al. 2013; Navratilova et al. 2015, 2012). Additionally, morphine administration did not alter MOR expression in these regions, suggesting that acute opioid exposure may not dynamically regulate MOR levels in these regions under the conditions tested. The lack of sex or strain differences in these regions may also explain the uniform antinociceptive effects of morphine across groups.

## 5 Conclusions

This study represents the first systematic analysis of sex‐ and strain‐specific differences in morphine‐induced behaviors and MOR expression in mice. Our findings highlight the complexity of sex and strain differences in opioid pharmacodynamics and emphasize the critical role of genetic background in shaping opioid responses. The stronger morphine‐induced hyperlocomotor activity seen in C57BL/6 mice suggests this strain is better suited for investigating the neural mechanisms of opioid side effects. The lack of strong behavioral sex differences in either strain suggests that research into the neurobiological sex differences in the processing of opioids may be best pursued using rats and people. Finally, our findings underscore the complexity of translating preclinical findings to broader biological principles and highlight the importance of considering species‐, strain‐, and sex‐specific factors when investigating opioid effects.

## Author Contributions

All authors had full access to all the data in the study and take responsibility for the integrity of the data and the accuracy of the data analysis. Conceptualization: L.J.M., D.C.B., S.K.R.; Methodology: L.J.M., D.C.B.; Investigation: D.C.B., S.K.R., M.F.; Formal analysis: D.C.B., S.K.R.; Resources: L.J.M.; Data curation: D.C.B., S.K.R.; Writing – original draft: D.C.B., S.K.R., M.F.; Writing – review and editing: L.J.M., D.C.B.; Visualization: D.C.B.; Supervision: L.J.M.; Funding acquisition: L.J.M.

## Conflicts of Interest

The authors declare no conflicts of interest.

### Peer Review

The peer review history for this article is available at https://www.webofscience.com/api/gateway/wos/peer‐review/10.1002/jnr.70039.

## Supporting information


Data S1.

Data S2.

Transparent Science Questionnaire for Authors

## Data Availability

The data that support the findings of this study are available from the corresponding author upon reasonable request.
