# Supplementary material for: Sex and Strain Differences in Analgesic and Hyperlocomotor Effects of Morphine and μ‐Opioid Receptor Expression in Mice
Source: J Neurosci Res. 2025 Apr 18;103(4):e70039. doi: 10.1002/jnr.70039 (PMC12006896; doi:10.1002/jnr.70039)
Supplement: Supplementary file 2 — Data S2. [file JNR-103-e70039-s002.pdf]

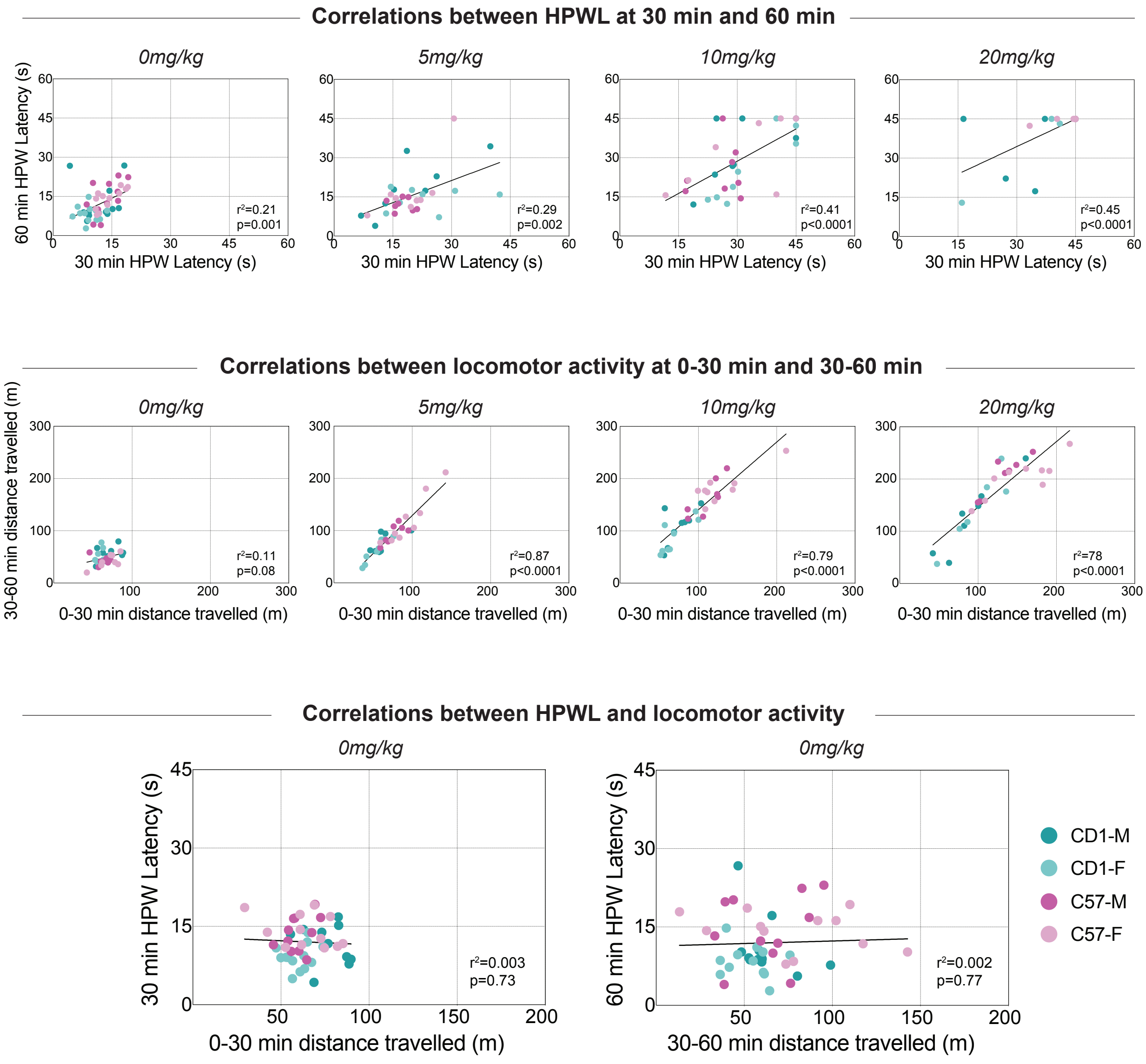

Supplementary Figure 1. Correlations between HPWL at the 30 min and 60 min tests indicates that each mouse showed consistency in their responses across these time points at all doses given (top row). Correlations between locomotor activity in the first 0-30 minutes and subsequent 30-60 minutes post injection show that only for mice given morphine demonstrate a strong relationship between these two time bins (middle row). Correlations between HPWL and locomotor activity at the 0mg/kg dose (i.e., saline) show no relationship, indicating that these two behaviours are generally independent of each other (bottom row). Simple linear regressions performed in GraphPad Prism.

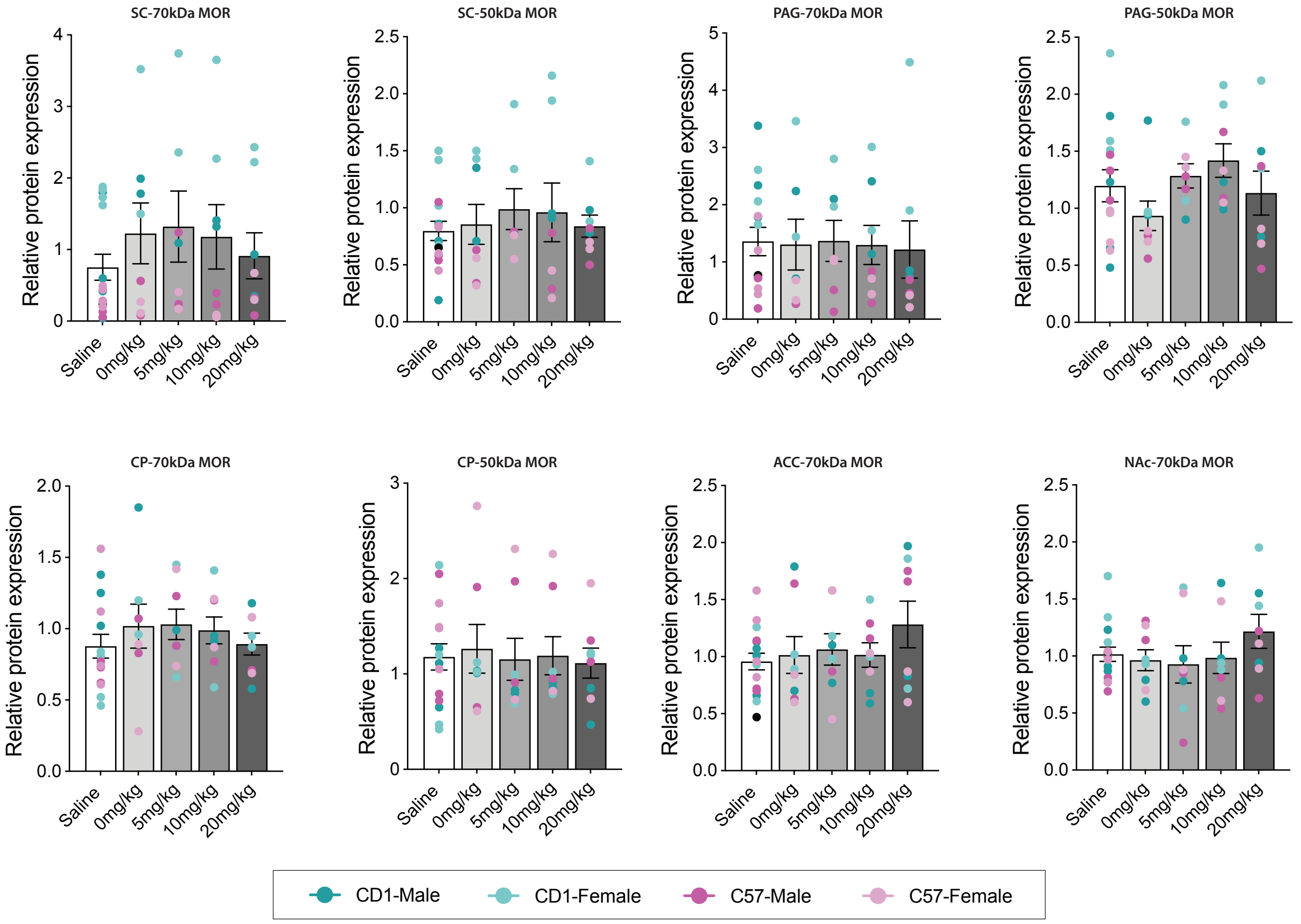

Supplementary Figure 2. The dose of morphine given on the final day (i.e., 24 hours before euthanasia and tissue collection) had no effect on MOR protein expression in either isoform in any of the regions, as determined by one-way ANOVAs. SC, spinal cord; PAG, periaqueductal gray; CP, caudatoputamen; ACC, anterior cingulate cortex; NAc, nucleus accumbens.

CD1(M) 13-24
CD1(F) 25-36

C57(M) 37-48
C57(F) 49-60

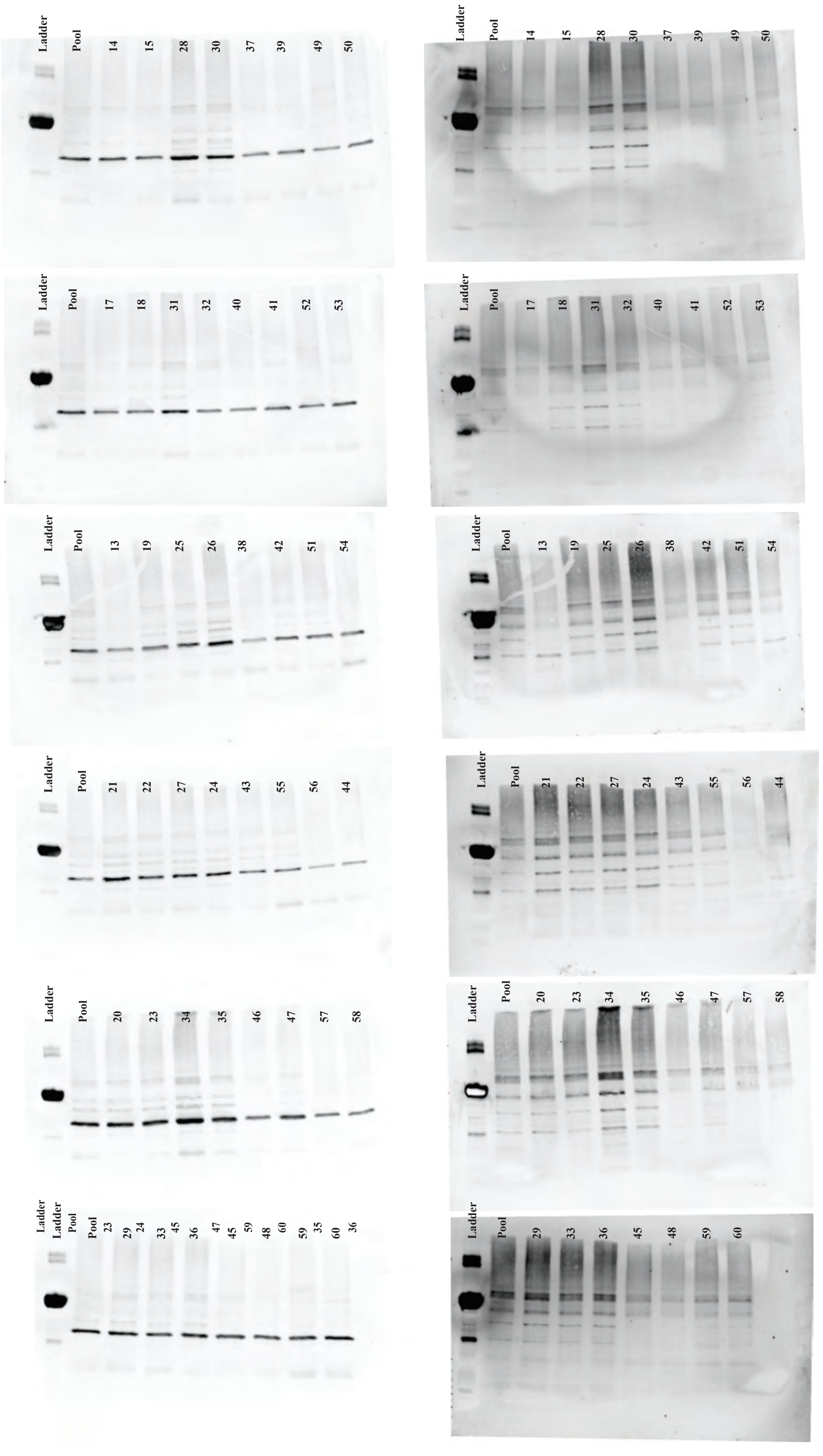

Raw western blots for beta actin (left column) and MOR (right column) in the Spinal cord (Figure 5a-c).

CD1(M) 13-24
CD1(F) 25-36

C57(M) 37-48
C57(F) 49-60

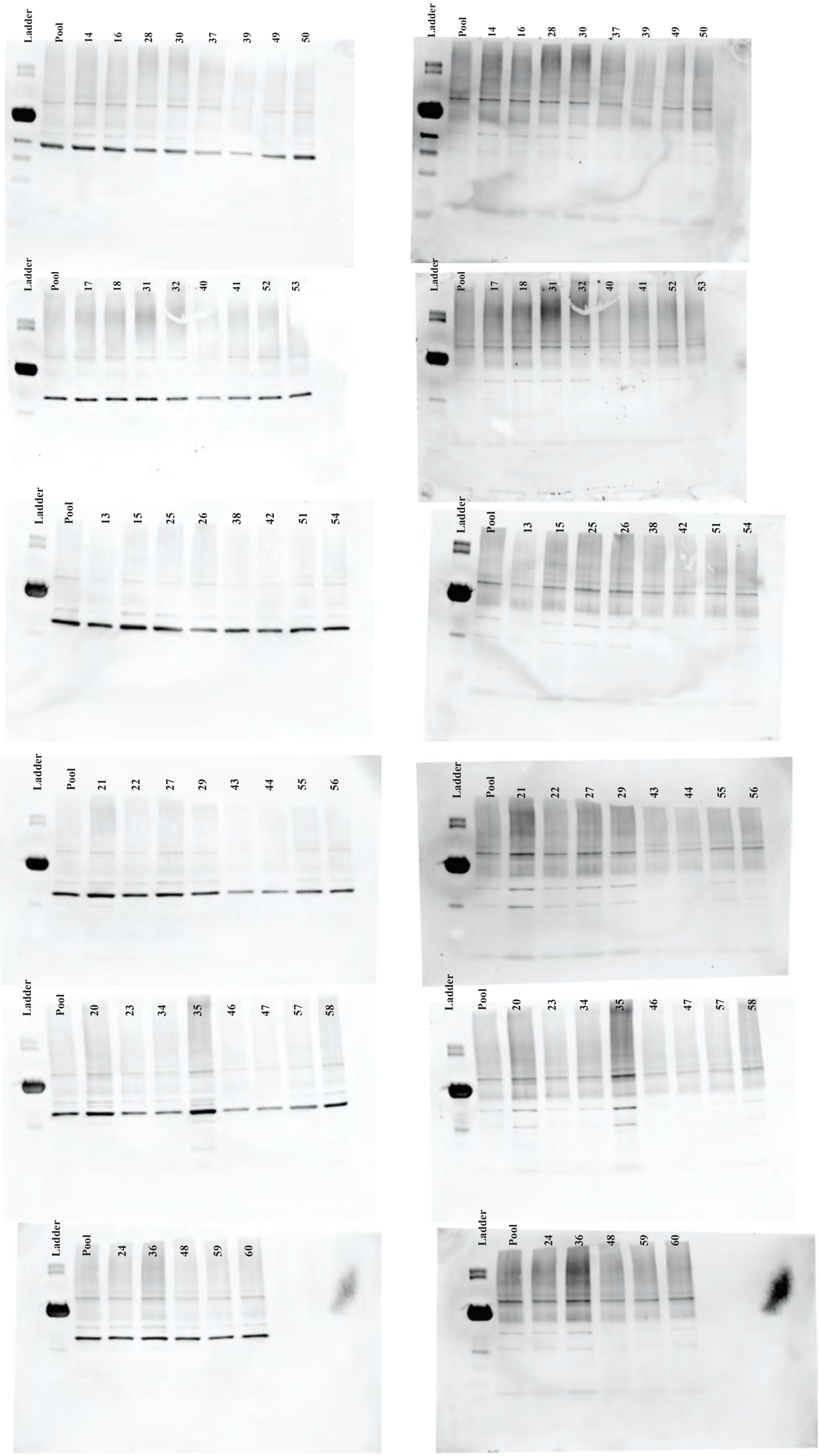

Raw western blots for beta actin (left column) and MOR (right column) in the periaqueductal gray (Figure 5d-f).

CD1(M) 13-24
CD1(F) 25-36

C57(M) 37-48
C57(F) 49-60

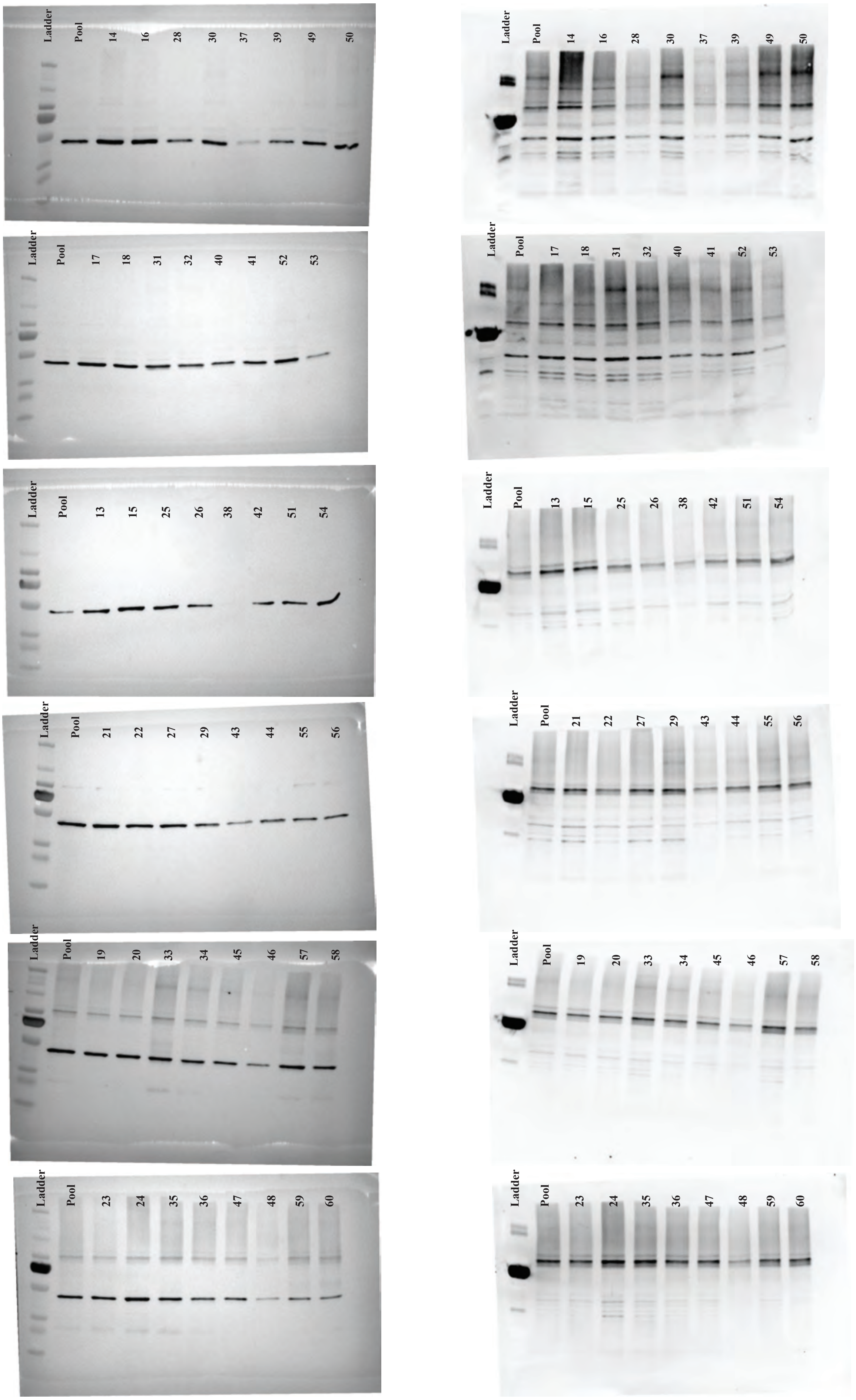

Raw western blots for beta actin (left column) and MOR (right column) in the caudate putamen (Figure 5g-i).

CD1(M) 13-24
CD1(F) 25-36

C57(M) 37-48
C57(F) 49-60

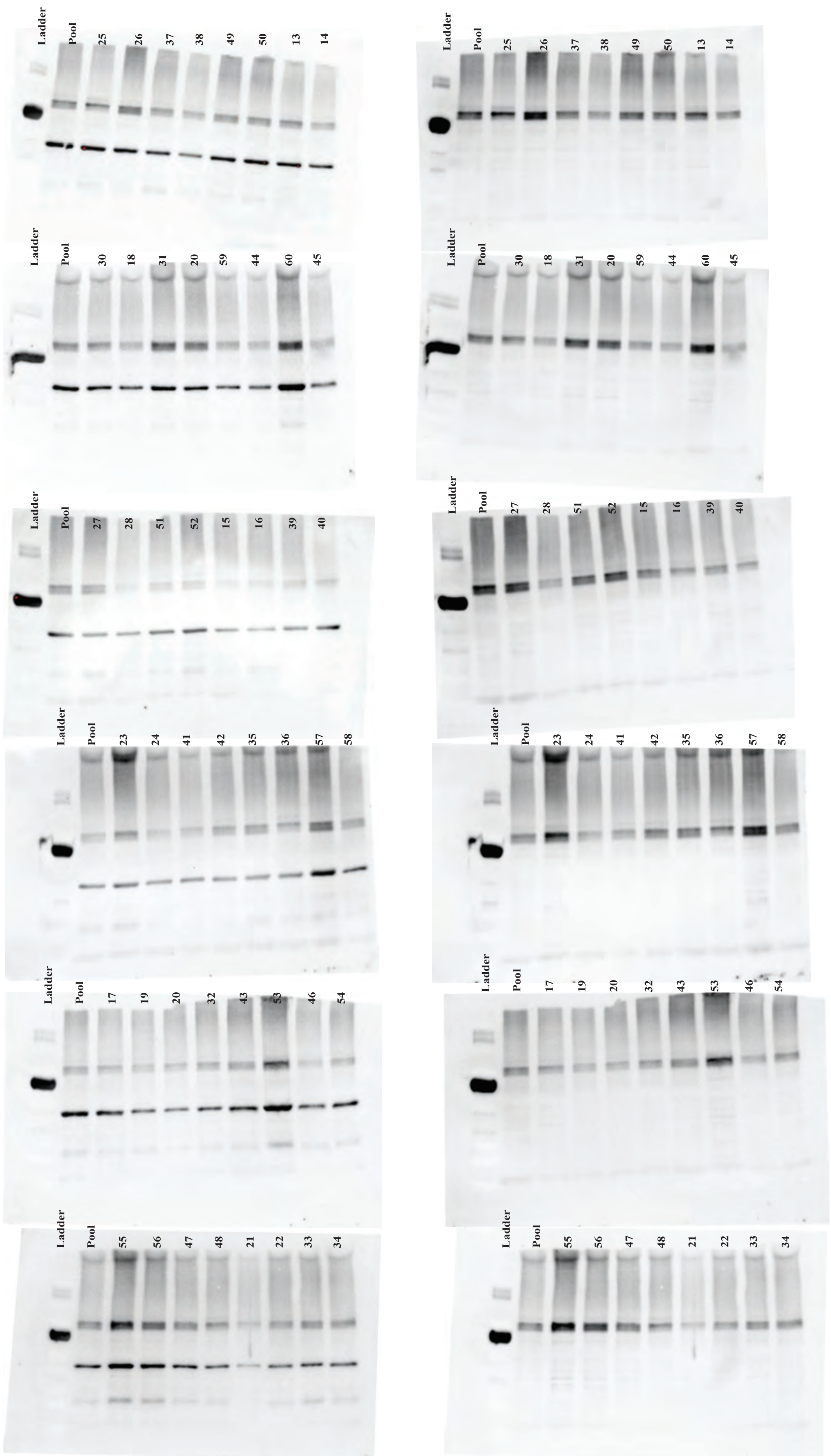

Raw western blots for beta actin (left column) and MOR (right column) in the nucleus accumbens (Figure 6a-c).

CD1(M) 13-24
CD1(F) 25-36

C57(M) 37-48
C57(F) 49-60

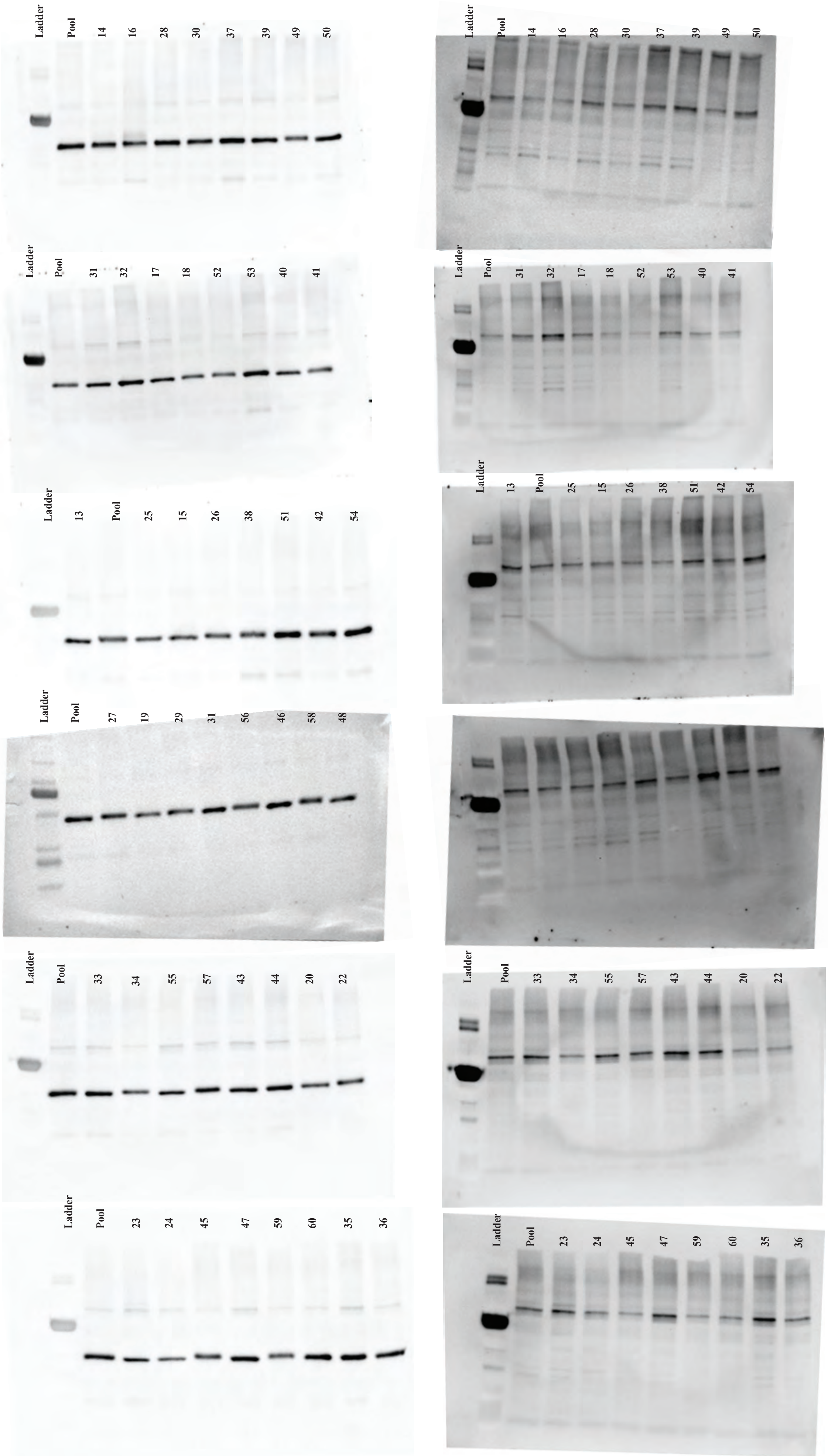

Raw western blots for beta actin (left column) and MOR (right column) in the anterior cingulate cortex (Figure 6d-f).
